# The effects of oxytocin and carbetocin on hemostatic parameters in women after spontaneous vaginal delivery: a randomized trial

**DOI:** 10.1590/1806-9282.20251346

**Published:** 2026-04-20

**Authors:** Nurcan Yoruk, Ayse Nur Aksoy, Berrin Göktug Kadioglu, Nazım Ozan Celebioglu, Konca Altinkaynak, Nayif Cicekli

**Affiliations:** 1 University of Health Sciences, Erzurum City Hospital, Department of Obstetrics and Gynecology – Erzurum, Türkiye.; 2 University of Health Sciences, Erzurum City Hospital, Department of Biochemistry – Erzurum, Türkiye.

**Keywords:** Carbetocin, Oxytocin, Labor, obstetric, Postpartum hemorrhage

## Abstract

**OBJECTIVE:**

This prospective randomized study compared the effects of two uterotonic drugs, oxytocin and carbetocin, on hemostatic parameters in patients undergoing spontaneous vaginal delivery.

**METHODS:**

One hundred women aged between 18 and 40 years, with term singleton pregnancies who underwent spontaneous vaginal delivery were enrolled in the study. Women with induced labor, preterm deliveries, multiple pregnancies, hypertension, macrosomia, polyhydramnios, and chronic disease were excluded. When women are admitted to the delivery room during the active phase of labor, eligible participants were randomly allocated in a 1:1 ratio to either oxytocin or carbetocin group. Following spontaneous vaginal delivery, the postpartum hemorrhage prophylaxis was provided with an intravenous infusion of 20 units of oxytocin in 1,000 mL ringer lactate solution for 2 h in the oxytocin group, and an intravenous infusion of 100 μg carbetocin in the carbetocin group. Maternal demographic and obstetric characteristics were recorded. Blood samples were collected just before vaginal delivery (T0), 2 h (T1) and 24 h (T2) following uterotonic infusion. Blood count, coagulation profile including D-dimer, fibrinogen, international normalized ratio, prothrombin time, and activated partial thromboplastin time were measured. Results were compared between groups using appropriate statistical tests.

**RESULTS:**

Serum levels of hemoglobin, platelet counts, prothrombin time, activated partial thromboplastin time, fibrinogen, D-dimer, and international normalized ratio measured at all time points were similar between groups (p>0.05). No serious side effects were observed in any patient.

**CONCLUSION:**

Slow infusion of oxytocin and carbetocin has comparable effects on hemostatic parameters with similar efficacy and with no clinically significant side effects.

## INTRODUCTION

Postpartum hemorrhage (PPH) is still the leading cause of maternal death worldwide, and it may occur after cesarean or vaginal delivery. The most important cause of PPH is ­uterine atony, and effective management of atony is important to reduce the risk of maternal mortality^1,2^.

Oxytocin, a neuropeptide hormone, is the first-line uterotonic agent. The administration of prophylactic oxytocin in the third stage of labor is recommended for the prevention of PPH^3^. A single intravenous (IV) administration of oxytocin may lead to serious hypotension. So, oxytocin is usually given as a continuous infusion in lactated Ringer's solution to prevent atony. But, oxytocin has a short half-life (4–10 min), and it reaches peak plasma levels within 30–60 min^4^. Carbetocin is an oxytocin analog with a longer half-life (40 min) than oxytocin^5^. In a study, comparing the effectiveness of prophylactic carbetocin with prophylactic oxytocin administrations in preventing PPH due to atony following vaginal delivery, similar efficacy and safety of both uterotonic agents were reported^6^.

As is known, pregnancy and postpartum period are characterized by an increased risk for venous thromboembolism and uterotonic drugs may further increase this risk. In a double-blinded randomized clinical study, researchers reported that oxytocin infusion leads to an increase in coagulability as measured by thromboelastography. They also reported that an increase in IV infusion of carbetocin dose from 15 to 30 IU/h increases coagulability further. They thought that oxytocin affects platelet function and the formation of fibrin from fibrinogen^7^.

We hypothesized that carbetocin, due to its long-lasting effect, may affect hemostatic parameters and increase coagulopathy more than oxytocin in the postpartum period. So, this study was planned to compare the effects of two uterotonic drugs, oxytocin and carbetocin, which have similar efficacy in preventing PPH, on hemostatic parameters and, therefore, the tendency to thromboembolism in patients undergoing spontaneous vaginal delivery.

## METHODS

This prospective randomized study was approved by the Ethical Committee of Erzurum City Hospital, Erzurum, Turkey (Project number: B.30.2.ATA.0.01.00/232). This study was conducted at the Obstetrics Clinic of Erzurum City Hospital between May 2024 and July 2024 in accordance with the Declaration of Helsinki, and written informed consent was obtained from all participants.

One hundred women aged between 18 and 40 years, with term singleton pregnancies who underwent spontaneous vaginal delivery were enrolled in the study. Participants were excluded if they had induced labor, preterm deliveries, multiple pregnancies, hypertension, preeclampsia, eclampsia, macrosomia, polyhydramnios, or chronic disease. Women with risk factors for developing atony, such as operative vaginal delivery, ­cesarean delivery, coagulopathy, trauma, and placental location or adhesion problems, and women receiving anticoagulant ­therapy were excluded. Cases of intrapartum fetal death as well as early neonatal death were also excluded from the study.

When women are admitted to the delivery room during the active phase of labor, eligible participants who agreed to take part in the study were randomly allocated in a 1:1 ratio to either oxytocin or carbetocin group. Women were ­randomized using computer generated random numbers by an independent researcher. Following spontaneous vaginal delivery, all women received active management of the third stage of labor (removal of the placenta by controlled cord traction, uterine massage and uterotonics). The PPH prophylaxis was provided with an IV infusion of 20 units of oxytocin (Synpitan forte®, Deva, Turkey) in 1,000 mL ringer lactate solution for 2 h in the oxytocin group^8^, and an IV infusion of 100 μg carbetocin (Pabal®, Ferring GmbH, Kiel, Germany) for half an hour in the carbetocin group. Both drugs were administered by the study nurse according to the random list after the delivery of the baby. Maternal demographic and obstetric characteristics (age, gestational age, height, weight, gravida, and parity) were recorded. Body mass index (BMI) was calculated as weight in kilograms divided by the square of height in meters (kg/m^2^). Blood samples were collected just before vaginal delivery (T0), 2 h (T1) and 24 h (T2) following uterotonic infusion. All blood samples of 10 cc were collected from the antecubital vein into tubes without anticoagulant and centrifuged at 3,500 *g* for 15 min. The separated serum was stored at -80°C until the assay. Blood count, coagulation profile including D-dimer, fibrinogen, international normalized ratio (INR), prothrombin time (PT), and activated partial thromboplastin time (aPTT) were measured. Plasma D-dimer levels were measured with a latex-enhanced photometric immunoassay kit (Siemens, Marburg, Germany) via a CS2500 automatic coagulation analyzer (Sysmex, Kobe, Japan). The values were expressed as ng/mL.

The primary endpoint of the study was the change in serum D-dimer levels at 24 h postpartum. Calculation of the sample size was based on the Testa et al.'s study^8^ using G* Power sample size calculator^9^. They reported that mean serum D-dimer levels at 24 h postpartum were 2.30±0.24 in oxytocin group and 2.46±0.32 in carbetocin group. According to G*Power analysis results; forty patients in each group were needed with a power of 80%, α **error** 5%, and β **error** 4%. Considering potential dropouts, 94 patients, 47 patients in each group, were included in the study.

Statistical analyses were performed with the Statistical Package for the Social Sciences (SPSS.22; IBM SPSS Statistics for Windows, Version 22.0). The normality of variables was tested using Kolmogorov-Smirnov test. Since the data show a normal distribution, the demographic characteristics and hematological parameters were compared between the groups using the independent t-test. The results were presented as mean±standard deviation and p≤0.05 was considered statistically significant. Analysis of variance (ANOVA) test was used to analyze the repetitive measurements in intra-group ­comparisons. Pearson's correlation coefficient was used to determine the linear relationship between Ddimer levels and other coagulation parameters.

## RESULTS

**Figure 1:**
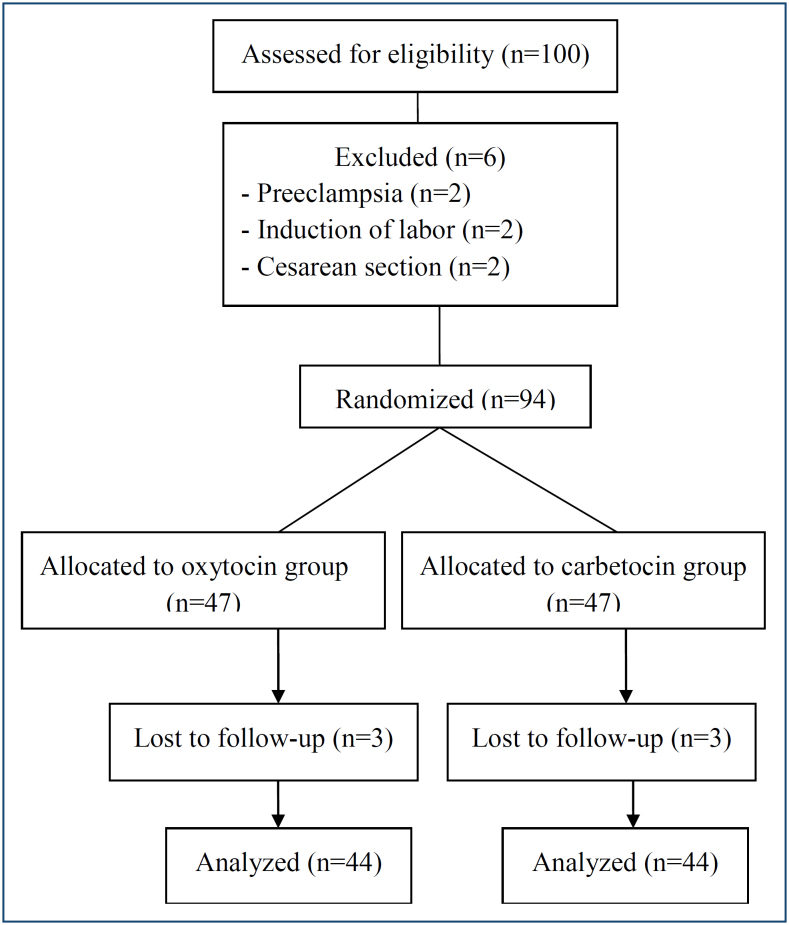
A flow chart of the study groups.

During study period, 100 patients were eligible, and 94 patients had inclusion criteria. All patients agreed to participate in the study. The study was completed with a total of 88 patients, including 44 patients in each group (Figure 1).

The demographic and obstetric characteristics were comparable among groups (Table 1). As seen in Table 2, serum levels of hemoglobin, platelet counts, PT, aPTT, fibrinogen, D-dimer, and INR measured at all time points were similar between groups (p>0.05). In comparison within groups, hemostatic parameters were not different among T_1_, T_2_, and T_3_ time points in both two groups (p>0.05). No correlation was found between serum D-dimer levels and maternal age (p=0.939, r=0.008). Also, there was no correlation between serum D-dimer levels and BMI (p=0.106, r=-0.174). However, there was a positive association between serum D-dimer and fibrinogen levels (p=0.023, r=0.242). No patient in either group had PPH requiring additional uterotonic medication. Also, tachycardia or hypotension requiring medical treatment was not observed in either group. No serious side effects were observed in any patient.

**Table 1:** The demographic and obstetric characteristics of the participants.

	Carbetocin group (n=44)	Oxytocin group (n=44)	p-value
Age (year)	26.8 (5.5)	28.0 (6.27)	0.362
Height (cm)	161.6 (6.4)	161.2 (5.6)	0.753
Weight (kg)	72.1 (10.6)	71.0 (9.8)	0.612
BMI (kg/m^2^)	27.5 (3.6)	27.3 (3.82)	0.762
Gravidity	6 (1.7)	5 (1.5)	0.766
Abortion	3 (0.3)	3 (0.3)	0.690
Gestational week	38.0 (2.7)	38.3 (1.6)	0.607

Values were expressed as mean (SD) or n (IQR). BMI: body mass index.

**Table 2:** Changes in hemostatic variables including hemoglobin, platelets, prothrombin time, activated partial thromboplastin time, D-Dimer, international normalized ratio, and fibrinogen values in groups at different time points.

		Carbetocin group (n=44)	Oxytocin group (n=44)	p-value
Hemoglobin (g/dL)				
	T0	12.3 (1.3)	12.1 (1.2)	0.427
	T1	12.1 (1.4)	12.0 (1.4)	0.807
	T2	11.2 (1.3)	11.1 (1.4)	0.934
PLTs (K/µL)				
	T0	243 (73.7)	239 (55.1)	0.781
	T1	242 (71.8)	232 (53.5)	0.446
	T2	219 (61.9)	221 (48.6)	0.846
aPTT (s)				
	T0	28.4 (2.9)	27.3 (4.3)	0.156
	T1	27.1 (4.3)	26.3 (3.90)	0.364
	T2	27.2 (2.8)	27.0 (3.58)	0.704
PT (s)				
	T0	12.8 (0.8)	13.0 (0.9)	0.404
	T1	13.2 (1.1)	12.8 (0.9)	0.084
	T2	12.9 (0.6)	12.8 (1.7)	0.815
D-Dimer (ng/mL)				
	T0	1,916.5 (912.1)	2,301.0 (1,161.6)	0.088
	T1	8,701.2 (10,016.7)	9,045.0 (9,722.1)	0.871
	T2	2,086.3 (1,372.9)	2,477.3 (2,070.2)	0.299
	(T2-T0)	169.8 (1,429.1)	193.9 (2,297.0)	0.953
INR				
	T0	0.9 (0.0)	0.9 (0.1)	0.520
	T1	1.0 (0.4)	0.9 (0.0)	0.148
	T2	0.9 (0.1)	0.9 (0.1)	0.426
Fibrinogen (mg/dL)				
	T0	485.0 (90.1)	473.0 (73.2)	0.493
	T1	460.5 (111.6)	456.7 (78.9)	0.856
	T2	468.8 (97.7)	449.7 (90.6)	0.344

Data were presented as mean (SD), INR: international normalized ratio, Hb: hemoglobin, PLT: platelets, PT: prothrombin time, aPTT: activated partial prothrombin time. Pre-treatment (T0), 2 h (T1) and 24 h (T2) after uterotonic infusion.

## DISCUSSION

This randomized study was designed to specifically compare the effect of carbetocin versus oxytocin on maternal hemosta tic parameters during spontaneous vaginal delivery. Both groups had similar serum levels of hemoglobin, platelet counts, PT, aPTT, fibrinogen, D-dimer, and INR after delivery. A similar action of both drugs on hemostatic parameters was observed.

Oxytocin, a uterotonic agent, is recommended as the first choice in PPH caused by atony^3^. However, due to its short half-life, oxytocin should be administered as a continuous IV infusion in PPH^4^. Carbetocin is a longer-acting uterotonic agent than oxytocin, and its effect begins rapidly and lasts for approximately 1 h^5^. Korb et al.^6^ reported similar effectiveness of prophylactic carbetocin with oxytocin for preventing severe PPH following vaginal delivery. Consistent with their results, participants who received carbetocin or oxytocin for PPH prophylaxis did not require additional uterotonics, and postpartum hemoglobin levels were similar in both groups in this current study. However, the material and methods of both studies are different. In the current study, patients who received labor induction were excluded. Whereas, patients who received labor induction and had an interventional delivery were included in the Korb et al's study^6^.

Pregnancy is associated with hemostatic system changes that generally lead to a state of hypercoagulability. These hemostatic changes include increased plasma levels of clotting factors, decreased endogenous anticoagulants, and fibrinolysis inhibition^10^. These changes are important in the maintenance of placental function and reducing postpartum bleeding. However, they may also contribute to an increased risk of thromboembolism, particularly toward the end of pregnancy and during puerperium^11^. D-dimer, a fibrin destruction product reflects ongoing coagulation and fibrinolysis. A significant association was reported between elevated serum D-dimer levels and an increased risk of incident venous thromboembolism (VTE) in the non-pregnant population^12,13^. Also, D-dimer elevation was reported to be associated with the risk of first VTE occurrence, VTE recurrence, and mortality^14^. Oxytocin and carbetocin, two effective uterotonic drugs used to prevent PPH, are thought to have unfavorable effects on hemostatic parameters. In a double-blinded clinical study, it was reported that oxytocin infusion at a rate of 30 IU/h in comparison to 15 IU/h causes a significant increase in coagulability^7^.

Testa et al.^8^ compared the effects of oxytocin and carbetocin on hemostatic system in patients undergoing elective cesarean section. In this observational study, blood samples for coagulation profile were collected before delivery, 1 and 24 h after drug infusion. They reported similar hemoglobin concentration, platelet count and coagulation profile between groups. Also, they reported a similar action of thrombin activation in both groups. They suggested that both carbetocin and oxytocin have similar effects on the coagulation system. Consistent with the results of this study, we observed similar serum levels of hemoglobin, platelet counts, PT, aPTT, fibrinogen, D-dimer, and INR in patients undergoing spontaneous vaginal ­delivery who received carbetocin or oxytocin for PPH prophylaxis.

In a recent randomized study^15^, Göksu and Karadeniz compared the effect of IV five units of oxytocin and 100 μg of carbetocin on the prevention of PPH in patients undergoing vaginal delivery with at least one risk factor for atony. They reported no significant differences between groups in terms of estimated bleeding volumes, additional uterotonic requirements, and hemoglobin decrease. In a prospective, randomized double-blind controlled study, Liu et al.^16^ also reported that prophylactic 100 μg IV infusion of carbetocin was not better than or 10 IU IV infusion of oxytocin to reduce the risk of PPH during vaginal delivery in high-risk women. These ­findings are consistent with the lack of difference in postpartum hemoglobin levels between the oxytocin and carbetocin groups in the current study. Conversely, Vernekar et al.^17^ reported slightly lower postpartum hemoglobin levels and a higher drop in hemoglobin levels in women received heat-stable carbetocin 100 μg intramuscular (IM) compared to the women received oxytocin 10 IU IM. They suggested that oxytocin is more effective in preventing PPH in women undergoing vaginal delivery. In another study^18^, carbetocin emerged as a superior option to oxytocin for PPH prophylaxis following vaginal delivery in high-risk singleton pregnancies. The reason for the conflicting results may be related to differences in patient selection and the route of administration of oxytocin in the studies.

To date, many studies comparing the effectiveness of oxytocin and carbetocin for the prevention of PPH have been carried out^15-18^. To our knowledge, this is the first study investigating the hemostatic effects of oxytocin and carbetocin in women undergoing spontaneous vaginal delivery. To minimize the cardiovascular side effects of oxytocin or carbetocin, both drugs are given as slow IV infusion for PPH prophylaxis in our clinical practice. This is a limitation of the current study. The effects of rapid and slow infusion of both drugs on hemostatic parameters could be investigated. Furthermore, the effects of different doses of oxytocin on hemostatic parameters could be compared. Another limitation is the exclusion of patients with risk factors for PPH. This may affect the results in comparing the effectiveness of both drugs in preventing PPH. However, it does not change their effects on hemostatic parameters.

In conclusion, slow infusion of oxytocin and carbetocin have comparable effects on the hemostatic parameters in patients undergoing spontaneous vaginal delivery. Comprehensive studies including larger patient groups are recommended to confirm these results.

## Data Availability

The datasets generated and/or analyzed during the current study are available from the corresponding author upon reasonable request.
